# Uric acid elevation in pediatric patients with dilated cardiomyopathy and prediction of mortality

**DOI:** 10.3389/fcvm.2024.1404755

**Published:** 2024-07-23

**Authors:** Yong Han, Cheng Chen, Suyuan Qin, Dongli Liu, Yusheng Pang

**Affiliations:** Department of Pediatrics, The First Affiliated Hospital of Guangxi Medical University, Nanning, China

**Keywords:** dilated cardiomyopathy, serum uric acid, mortality, heart failure, children

## Abstract

**Background and aims:**

Pediatric dilated cardiomyopathy (DCM) is a primary cause of heart failure, highlighting the urgent need for effective prognostic markers.

**Methods:**

We performed a single-center retrospective study involving 145 children diagnosed with DCM, with a median follow-up period of 4.0 months (interquartile range: 6.2–108.4 months). The relationship between serum uric acid (SUA) levels and all-cause mortality was assessed using Kaplan–Meier survival curves, multivariate Cox proportional hazard models, and restricted cubic spline (RCS) models.

**Results:**

Of the 145 children with DCM (median age 5.7 years; 61.4% male), 45 (31%) died within 1 year, and 65 (44.8%) died during the maximum follow-up period. In adjusted multivariate Cox regression models, each log2 SUA increase was linked to a higher risk of 1-year mortality [hazard ratio (HR), 2.66; 95% confidence interval (CI), 1.41–5.01] and overall mortality (HR, 1.97; 95% CI, 1.15–3.37). The highest SUA tertile showed a greater risk of mortality at 1 year (HR, 4.26; 95% CI: 1.5–12.06) and during the maximum follow-up (HR, 2.56; 95% CI: 1.06–6.16) compared with the lowest tertile. RCS models indicated an inverted L-shaped association between baseline SUA levels and overall mortality risk, with age-stratified analyses revealing a linear and U-shaped relationship in children ≤10 and >10 years, respectively. Further age-stratified analyses highlighted the modifying effect of age on this association.

**Conclusion:**

Elevated SUA levels are a significant predictor of mortality in pediatric DCM, with a pronounced impact on children under 10 years of age. Therefore, SUA levels could serve as potential biomarkers for risk stratification in this population.

## Introduction

Dilated cardiomyopathy (DCM) is a prevalent cause of heart failure (HF) in children (1, 2). Epidemiological data indicate that around 30%–40% of symptomatic pediatric patients either undergo heart transplantation or die within a year of diagnosis (3, 4). Previous studies have identified prognostic factors for pediatric DCM; however, no effective risk-stratification scheme has been developed (5, 6).

In humans, uric acid is the final oxidation product of purine metabolism. Clinical studies have shown a strong correlation between high serum uric acid (SUA) levels and both the severity and mortality of HF, establishing its recognized prognostic role in HF (7, 8). Additionally, managing hyperuricemia (HUA) has been linked to improved long-term outcomes in HF patients, potentially due to better vascular endothelial function (9). A recent Mendelian randomization study identified elevated SUA levels as a causal factor in the development of HF, emphasizing its potential as a therapeutic target (10). Although there is evidence supporting the association between SUA levels and cardiovascular disease in adults, it remains uncertain whether these findings can be applied to children. Understanding the relationship between SUA levels and cardiovascular outcomes in this critical demographic may offer opportunities for early intervention and improved prognosis. This study aims to investigate the relationship between SUA levels and mortality risk, and to determine its prognostic significance in children hospitalized with DCM.

## Methods

### Study population

We conducted a single-center retrospective analysis of children hospitalized for DCM at our institution between January 2012 and January 2023. Eligibility criteria included left ventricular dilation [left ventricular end-diastolic dimension (LVEDD) *Z*-score >2] and left ventricular systolic dysfunction (LVEF <55%) (5). Due to varying effects of SUA levels across different types of HF, we excluded patients with mixed DCM, such as hypertrophic, restrictive, or noncompaction cardiomyopathy. Our focus was on children with primary DCM (5); thus, we excluded those with congenital left-to-right shunt heart disease, myocarditis, coronary artery disease, or metabolic diseases. Infants younger than 1 month were excluded to eliminate the influence of maternal factors. Additionally, patients with severe kidney disease and those missing SUA levels were not included. The final cohort consisted of 145 pediatric patients hospitalized for DCM who met the inclusion criteria. The study was approved by the research ethics committee of the First Affiliated Hospital of Guangxi Medical University (approval number: 2023-E478-01). Informed consent was obtained from the parents or guardians of all participants.

### Clinical data and laboratory values

Baseline data were collected from initial hospitalization records, encompassing (1) demographics such as sex, age, and prior HF diagnosis; (2) physical examination findings at admission, including New York Heart Association (NYHA)/Ross classification and body mass index (BMI); (3) laboratory indicators and echocardiographic findings (Table 1); and (4) discharge medications, including renin-angiotensin-aldosterone system inhibitors, digoxin, diuretics, and β-blockers. For patients with multiple admissions, only data from the first admission were used to avoid confounding factors. Laboratory data were collected within 24 h, and echocardiography and electrocardiography data were gathered within 48 h of admission. The LVEDD was normalized using age- and body surface area-specific *Z*-scores. The LVEF was calculated from the parasternal long-axis view using the Teichholz method.

**Table 1:** Baseline characteristics of participants by tertile of serum uric acid.

Variables	Total	Serum uric acid			
		1st tertile (70–266 µmol/L)	2nd tertile (266–415 µmol/L)	3rd tertile (415–1,015 µmol/L)	*p*-value
Participants, *n*	145	48	48	49	
Age, year	5.7 (1.0, 11.6)	0.9 (0.5, 2.5)	6.2 (1.6, 10.8)	11.8 (7.4, 14.2)	**<0.001**
Sex, male, *n* (%)	89 (61.4)	27 (56.2)	31 (64.6)	31 (63.3)	0.666
Preexisting HF ≥6 months, *n* (%)	23 (15.9)	4 (8.3)	7 (14.6)	12 (24.5)	0.089
NYHA/ROSS class, *n* (%)					0.052
Ⅱ	96 (66.2)	36 (75)	34 (70.8)	26 (53.1)	
Ⅲ/Ⅳ	49 (33.8)	12 (25)	14 (29.2)	23 (46.9)	
BMI, kg/m^2^	16.0 ± 3.0	15.2 ± 2.6	15.9 ± 3.0	16.9 ± 3.2	**0** **.** **014**
**Laboratory values**					
SCr, µmol/L	44.8 ± 31.5	28.3 ± 13.2	42.0 ± 21.2	63.7 ± 41.5	**<0** **.** **001**
BUN, mmol/L	5.7 ± 2.8	4.2 ± 1.9	5.5 ± 2.0	7.3 ± 3.3	**<0.001**
Cystatin, mg/L	0.9 ± 0.2	0.9 ± 0.1	0.9 ± 0.2	1.0 ± 0.2	0.09
eGFR, ml/min/1.73 m^2^	80.0 ± 14.0	82.3 ± 13.2	80.2 ± 12.6	77.5 ± 15.9	0.244
Serum potassium, mmol/L	4.3 ± 0.6	4.4 ± 0.6	4.3 ± 0.6	4.3 ± 0.8	0.794
**Echocardiography findings**					
LVEDD *z*-score	6.9 ± 2.2	7.3 ± 2.6	6.7 ± 2.0	6.6 ± 1.9	0.289
LVEF, %	31.6 ± 9.9	33.3 ± 8.5	33.0 ± 10.3	28.6 ± 10.3	**0** **.** **034**
**Discharge medication**					
RASSi, *n* (%)	121 (91.7)	38 (86.4)	39 (90.7)	44 (97.8)	0.121
Digoxin, *n* (%)	114 (86.4)	37 (84.1)	41 (95.3)	36 (80)	0.096
Diuretics, *n* (%)	125 (94.7)	42 (95.5)	40 (93)	43 (95.6)	0.793
β-blocker, *n* (%)	97 (73.5)	30 (68.2)	34 (79.1)	33 (73.3)	0.516

Data presented are the mean ± SD, median (Q1–Q3), or *N* (%).

BMI, body mass index; NYHA, New York Heart Association; SCr, serum creatinine; BUN, blood urea nitrogen; eGFR, estimated glomerular filtration rate; LVEDD, *z*-score left ventricular end diastolic diameter data were recorded and normalized into *Z* scores using age and body surface area; LVEF, left ventricular ejection fractions; RASSi, renin-angiotensin-aldosterone system inhibitors.

*P* < 0.05 were shown in bold.

### Study outcome

The primary outcome was all-cause mortality or heart transplantation, assessed at 1 year and during long-term follow-up (maximum duration: 108.4 months). Follow-ups included telephone interviews and regular outpatient clinic visits. Information on deaths was also obtained from provincial registries for children lost to follow-up.

### Statistical analysis

Participants were categorized into tertiles based on their SUA levels. Baseline characteristics were detailed according to these tertiles. Group differences were evaluated using statistical tests appropriate for the data type. Specifically, categorical variables were assessed with the chi-squared test, normally distributed variables with one-way analysis of variance, and skewed variables with Kruskal–Wallis tests. Characteristics are presented as mean ± standard deviation, median with interquartile range (IQR), or number (*n*) with percentage (%), as suitable. Kaplan–Meier cumulative incidence curves were created for SUA level tertiles and compared using the log-rank test.

To examine the association between SUA levels and all-cause mortality at 1 year and maximum follow-up, as well as the dose-response relationship, we used multivariate Cox regression models and restricted cubic spline (RCS) models. Covariates for the regression model were chosen based on their clinical relevance and univariate analysis results (Supplementary Tables S1, S2). Before multivariate analysis, significant univariate variables were checked for collinearity using a tolerance cutoff of <0.02 and a variance inflation factor cutoff of >5 (Supplementary Tables S3). Non-collinear variables were included in the multiple regression model and reported as hazard ratios (HRs) with 95% confidence intervals (CIs) and corresponding *p*-values.

Three multivariate Cox regression models were adjusted for different covariates: Model 1 was unadjusted; Model 2 was adjusted for age and sex; and Model 3 was adjusted for Model 2 plus BMI, serum creatinine (SCr), blood urea nitrogen (BUN) levels, and LVEF. SUA levels were analyzed as continuous or categorical variables in tertiles. When treated as a continuous variable, SUA levels were log-transformed (base 2) to minimize the influence of outliers, and the data were presented as per log2 unit increase. Multivariate Cox regression models were constructed using the baseline SUA tertiles, with the lowest SUA tertile as the reference group. Variables with more than 10% missing values were excluded, while those with less than 10% missing values were imputed using the mean.

Subgroup analyses were stratified by relevant covariates, including sex, age, NYHA/Ross score, LVEF, and estimated glomerular filtration rate (eGFR). A cutoff age of 10 years was used for stratification as it represents the onset of adolescence. Interactions among subgroups were assessed using likelihood ratio (LR) tests.

All statistical analyses were conducted using SPSS (version 27.0; IBM Inc., New York, NY, USA) and R Statistical Software version 4.2.0 (http://www.R-project.org; R Foundation).

## Results

### Baseline characteristics of participants

We analyzed data from 145 patients with a median age of 5.7 years [interquartile range (IQR): 1.0–11.6]. Among these patients, 89 (61.4%) were male, and 56 (38.6%) were female. Forty-nine patients (33.8%) exhibited NYHA class III or IV symptoms, and 25 (15.9%) had a history of HF. Patients in the highest SUA tertile were generally younger and had worse SCr, BUN levels, and LVEF at admission (*P* < 0.05). The types of medications prescribed at discharge were similar across the tertiles, with digoxin, diuretics, and RAASi being more commonly used, while β-blockers were mostly prescribed post-discharge. Table 1 outlines the participants' baseline characteristics and their associations with SUA tertiles.

### Distribution of baseline SUA levels

**Figure 1:**
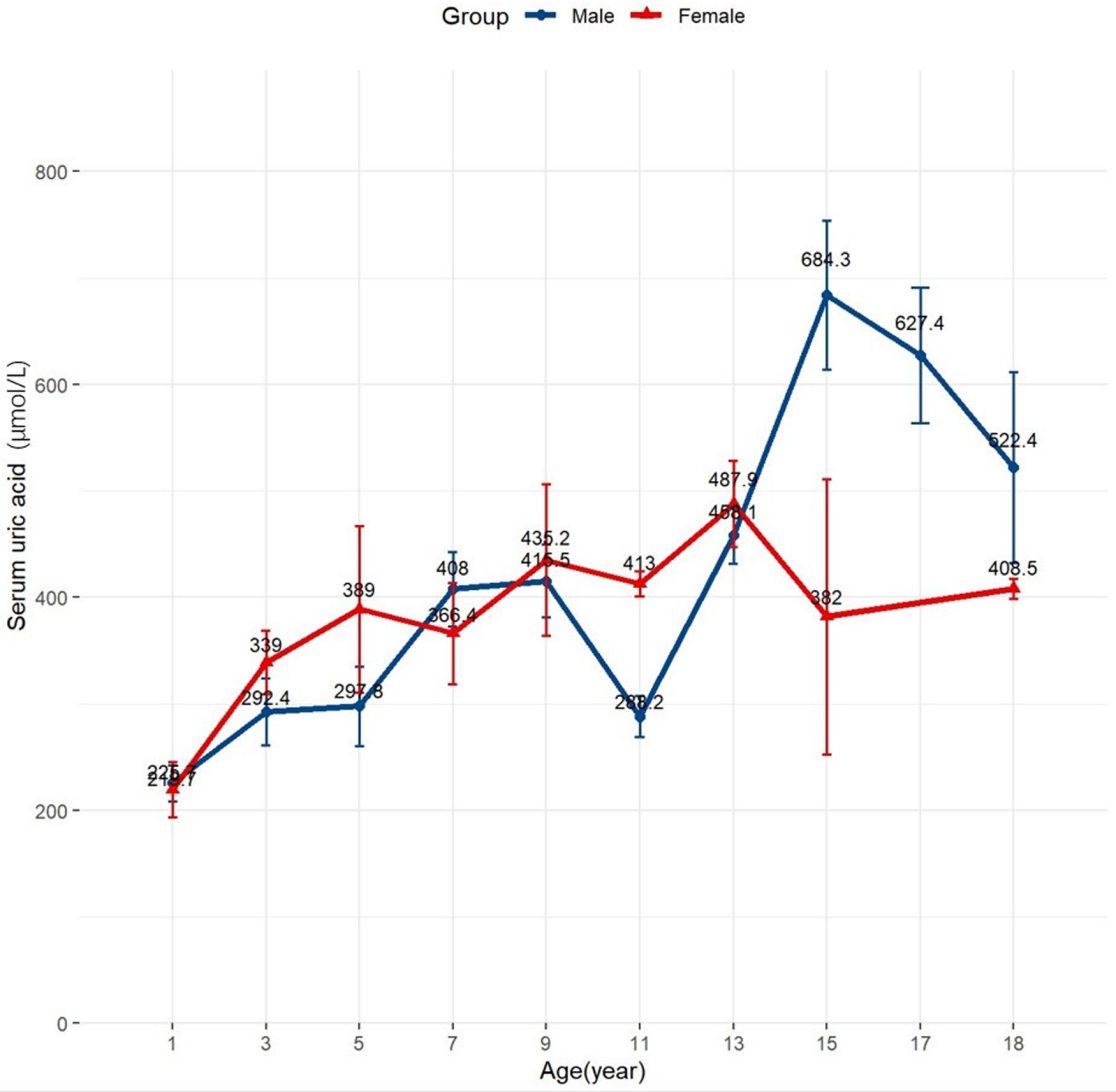
Distribution of baseline uric acid values in the study population. Changes of mean baseline uric acid levels according to age and sex.

As shown in Table 1, the SUA tertiles were categorized as follows: lowest (1st tertile) 70–266 µmol/L, median (2nd tertile) 266–415 µmol/L, and highest (3rd tertile) 415–1,015 µmol/L. Figure 1 illustrates that SUA levels varied by age and sex. In children under 10 years, the mean SUA levels increased almost linearly with no significant differences between sexes. However, in males aged 10 years and older, SUA levels rose rapidly with considerable fluctuations, while in females, the mean SUA levels plateaued around age 13 and then declined.

### Endpoint event rates stratified by baseline SUA levels

**Figure 2:**
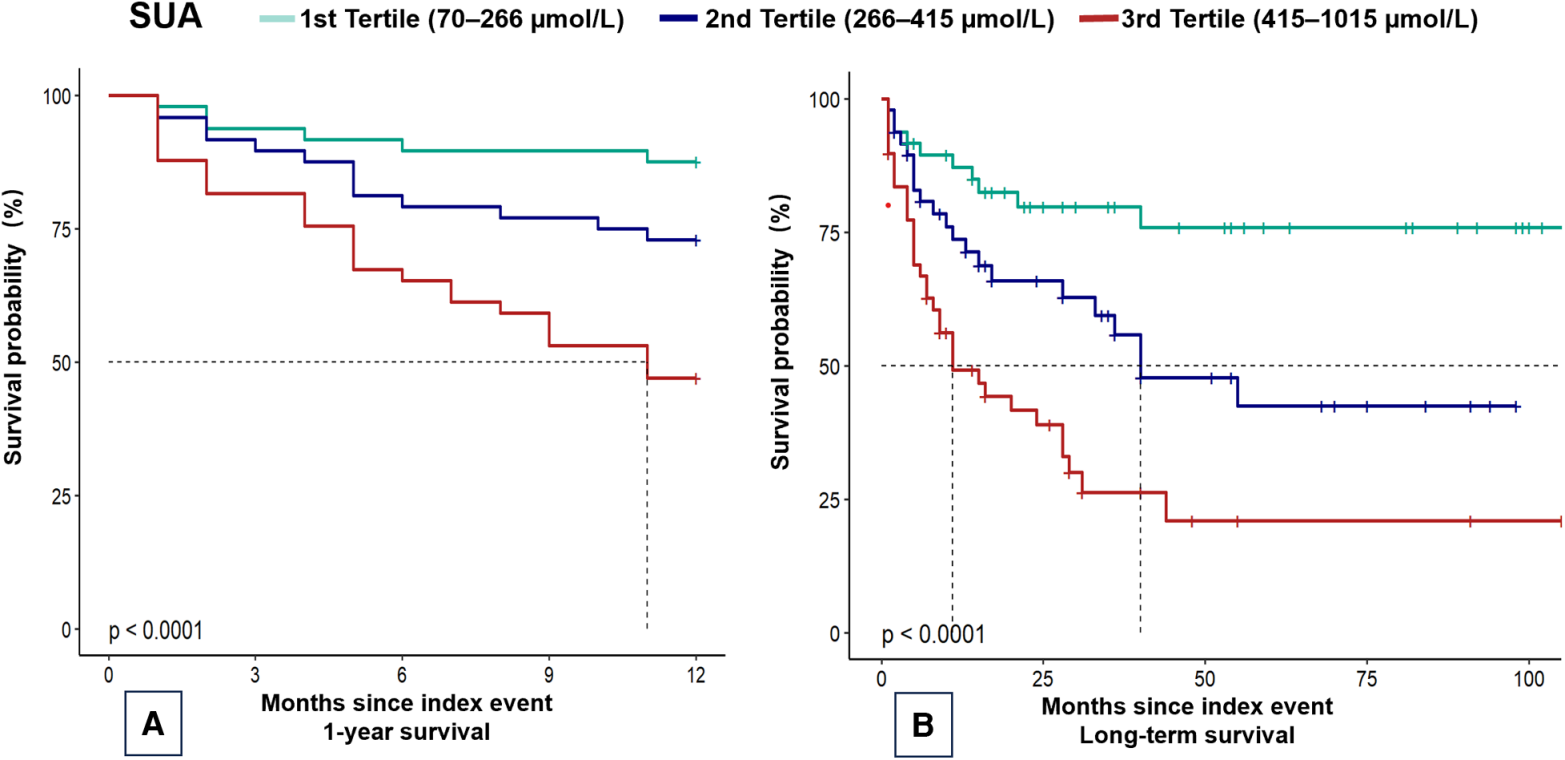
Kaplan–Meier plots of mortality in children with dilated cardiomyopathy. Kaplan–Meier curves for (**A**) 1-year mortality and (**B**) overall mortality. SUA, serum uric acid.

In this cohort of 145 patients, the median follow-up period was 4.0 months (IQR: 6.2–108.4 months). The all-cause mortality rate was 31% (45 patients) at 1 year and 44.8% (65 patients) at the maximum follow-up. None of the patients underwent heart transplantation. Kaplan–Meier estimates for 1-year and maximum follow-up periods revealed significant differences in the incidence of all-cause mortality among the SUA tertiles during the follow-up period (*P* < 0.01) (Figures 2A,B).

### Association between baseline SUA levels and outcomes

To identify independent predictors of mortality risk and analyze the dose-response relationship, multivariate Cox regression analyses were conducted (Table 2). After adjusting for age, sex, BMI, SCr and BUN levels, and LVEF, SUA levels were found to be independently associated with mortality at 1 year (HR = 2.66 per log2 unit increase) and during the maximum follow-up period (HR = 1.97 per log2 unit increase). Compared to the lowest SUA tertile, the highest SUA tertile was linked to a higher incidence of all-cause mortality at 1 year (HR = 4.26, 95% CI: 1.5–12.06) and during the maximum follow-up (HR = 2.56, 95% CI: 1.06–6.16), after adjusting for age, sex, BMI, SCr, BUN, and LVEF (Table 1). A dose-response relationship was evident in each model (*P* for trend < 0.05). No significant differences in all-cause mortality were found between the lowest and median SUA tertiles.

**Table 2:** 1-year mortality and long-term mortality associated with baseline SUA in DCM.

Outcome	Model 1		Model 2		Model 3	
	HR_95% CI	*P*-value	HR_95% CI	*P*-value	HR_95% CI	*P*-value
**SUA per log2 unit increase (µmol/L)**						
1-year mortality	2.78 (1.76–4.39)	<0.001	2.99 (1.63–5.45)	<0.001	2.66 (1.41–5.01)	0.003
Overall mortality	3.05 (2.04–4.55)	<0.001	2.31 (1.4–3.82)	0.001	1.97 (1.15–3.37)	0.014
**SUA as a categorical variable**						
1-year mortality						
1st tertile	1 (Ref)		1 (Ref)		1 (Ref)	
2nd tertile	2.35 (0.89–6.18)	0.084	2.21 (0.81–6.06)	0.122	1.99 (0.72–5.48)	0.183
3rd tertile	5.44 (2.24–13.25)	<0.001	5.07 (1.84–13.98)	0.002	4.26 (1.5–12.06)	0.006
*P* for trend		<0.001		0.001		0.004
Overall mortality						
1st tertile	1 (Ref)		1 (Ref)		1 (Ref)	
2nd tertile	2.51 (1.18–5.34)	0.017	1.83 (0.82–4.08)	0.14	1.8 (0.8–4.04)	0.154
3rd tertile	5.21 (2.55–10.64)	<0.001	3.14 (1.36–7.25)	0.007	2.56 (1.06–6.16)	0.036
*P* for trend		<0.001		0.005		0.036

Model 1 was a crude model that was unadjusted; Model 2 was adjusted for age and sex; Model 3 was adjusted for Model 2 as well as BMI, SCr, BUN and LVEF.

Ref, reference; DCM, dilated cardiomyopathy; SUA, serum uric acid; BMI, body mass index; SCr, serum creatinine; BUN, blood urea nitrogen; LVEF, left ventricular ejection fractions; HR, hazard ratio; CI, confidence index.

**Figure 3:**
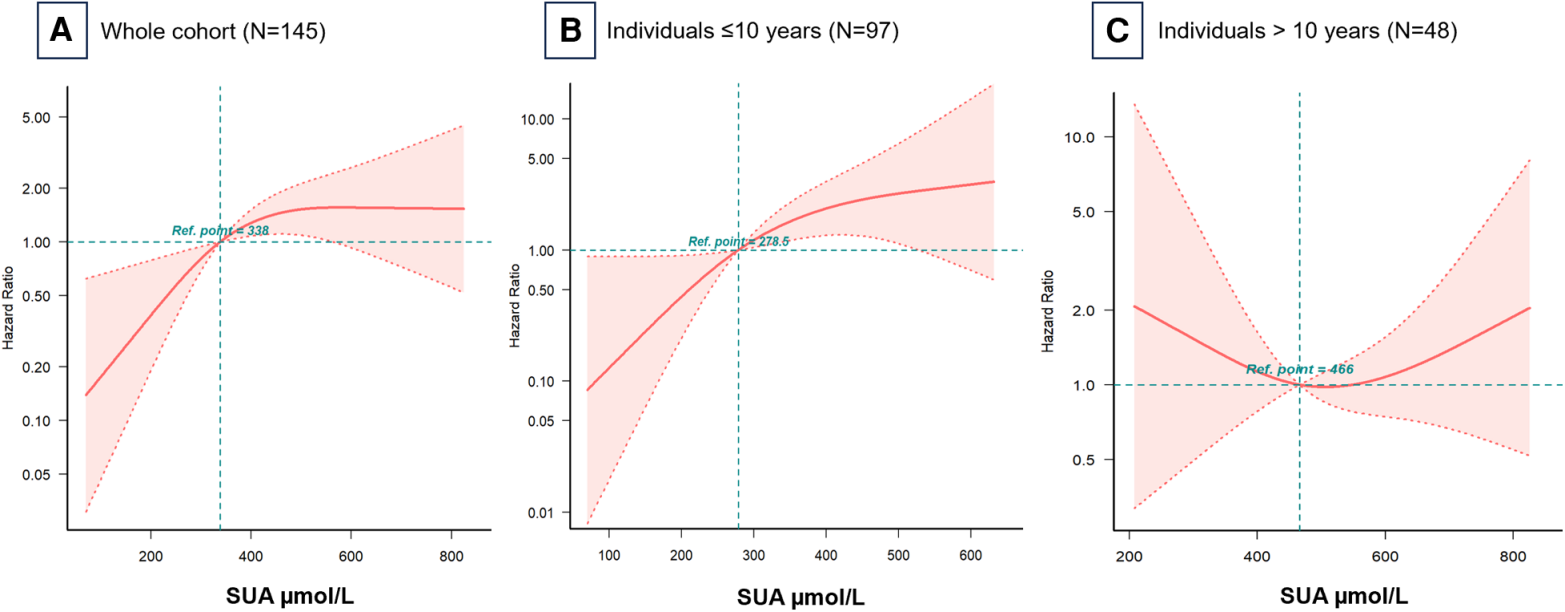
Hazard ratios for all-cause mortality based on baseline serum uric acid levels. Restricted cubic spline models for (**A**) the whole cohort, (**B**) individuals ≤10 years, and (**C**) individuals >10 years. Adjusted for all covariates in Table 2 Model 3. Ref, reference; SUA, serum uric acid.

Figure 3A illustrates the RCS models, which revealed an inverted L-shaped association between baseline SUA levels and all-cause mortality risk. Further age-stratified analysis in 10-year intervals showed notable differences in the curve trajectories (Figures 3B,C). For individuals under 10 years old, a clear linear relationship was observed, with SUA levels up to 278.5 μmol/L correlating with a higher risk of mortality. In contrast, for individuals aged 10 years and older, the relationship between SUA and mortality approximated a U-shaped curve, with the lowest risk of all-cause mortality occurring at an SUA of 466 μmol/L.

### Subgroup analysis

**Figure 4:**
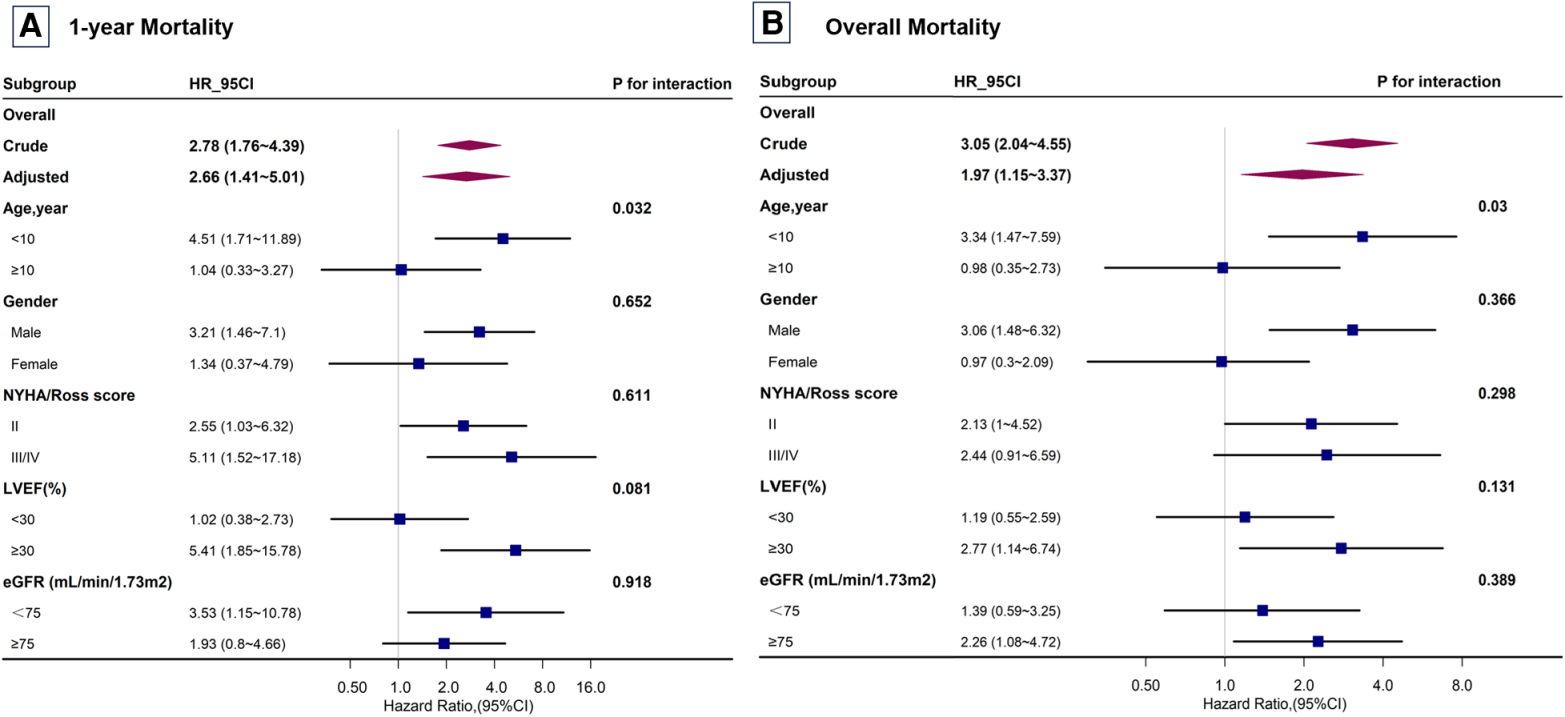
Subgroup analysis of the risk of mortality in children with dilated cardiomyopathy. Subgroup analysis for (**A**) 1-year mortality and (**B**) overall mortality. Adjusted for all covariates in Table 2 Model 3. NYHA, New York Heart Association; LVEF, left ventricular ejection fraction; eGFR, estimated glomerular filtration rate.

In the subgroup analysis stratified by sex, age, NYHA/Ross score, LVEF, and eGFR, each log2 unit increase in SUA concentration showed a significant age-related modification effect (*P* < 0.05) (Figures 4A,B). Interactions across the subgroups were tested using the likelihood ratio test. Among participants younger than 10 years, each log2 unit increase in SUA concentration significantly increased the risk of mortality. However, for participants older than 10 years, an increase in SUA concentrations did not significantly reflect this risk.

## Discussion

Our study is the first to examine the relationship between baseline SUA levels and the risk of all-cause mortality in children with DCM. The results show that elevated SUA levels are a significant and independent risk factor for all-cause mortality in pediatric patients, even after adjusting for age, gender, kidney function, and LVEF in Cox regression models. Age-stratified analyses revealed that this association is influenced by age, with elevated SUA showing a positive linear relationship with increased mortality risk in younger children (aged ≤10 years). However, elevated SUA did not significantly impact mortality risk in adolescents (aged 10–18 years).

These findings align with previous research on the relationship between SUA levels and prognosis in specific cardiovascular diseases among children. Hsia et al. analyzed data from 13,241 pediatric patients, finding that progressively higher SUA levels were associated with increased all-cause and cardiovascular mortality (11). Compared to SUA levels below 6.0 mg/dl, SUA levels of 6.0–8.9, 9.0–11.9, and ≥12.0 mg/dl had stepwise adjusted HRs for mortality of 1.02 (95% CI: 0.82–1.26), 1.48 (95% CI: 1.08–2.02), and 4.73 (95% CI: 2.67–8.37), respectively. Additionally, HUA was linked to a higher risk of mortality from cardiovascular disease (HR = 5.0, 95% CI: 1.79–13.94). Li et al. also found that elevated SUA levels in children with DCM were correlated with NYHA functional class and echocardiographic parameters (12).

In our study, the highest SUA tertiles showed more severe renal function impairment. This finding is consistent with the established link between elevated SUA levels and renal dysfunction (13, 14). However, whether SUA independently contributes to the development and progression of HF or merely reflects the extent of renal dysfunction and chronic kidney disease is still under investigation. Unlike adults, pediatric patients with HF rarely exhibit comorbidities such as obesity, hypertension, diabetes, and renal insufficiency, which usually manifest later in life. Nonetheless, our findings in children mirror those observed in adults with HF (7, 15–17), suggesting that SUA levels may play a direct role in the pathophysiology of HF. In HF, HUA may result from elevated xanthine oxidase activity induced by inflammatory cytokines and oxidative stress (18, 19). Alternatively, it could be due to increased renal tubular reabsorption coupled with decreased urate excretion, or a combination of these mechanisms (20, 21).

Our extended analyses revealed an age-dependent relationship between SUA levels and mortality risk in children with DCM. Younger children (aged ≤10 years) appear more susceptible to the effects of uric acid compared to those older than 10 years. This indicates that age is a crucial factor influencing how SUA levels affect the prognosis of pediatric DCM. The interaction between SUA levels and age may be related to variations in SUA levels among children (Figure 1). These variations could be driven by changes in pubertal sex hormones; testosterone increases uric acid levels by promoting muscle anabolism and reducing excretion, while estrogen promotes uric acid excretion (22, 23). Racial differences in the expression of renal urate transporters and higher renal urate reabsorption in males compared to females might also contribute to the observed sex differences in SUA levels (23). Individual factors such as age and sex might differently impact the association between SUA levels and mortality risk in children with DCM. However, our study did not find a significant interaction between sex and SUA levels, which may be due to the limited sample size.

Emerging evidence suggests that lowering SUA levels could improve patient outcomes. However, the current research remains inconclusive about whether such reductions offer meaningful clinical benefits. Some studies in adults have reported no significant effect of SUA-lowering treatments on HF patients (24–27), indicating the need for further investigation. The potential advantages of treating HUA in pediatric HF patients remain unexplored. Although our findings indicate that baseline SUA levels have independent prognostic importance, further research is necessary to explore the impact of other potential confounding variables not included in this study, such as the use of diuretics and allopurinol. Moreover, we lacked genetic and SNP (single nucleotide polymorphism) data, which limited our ability to conduct further analyses. Recent Mendelian randomization studies, which rely on genetic information, have provided compelling evidence for a causal relationship between SUA and HF, underscoring the importance of incorporating genetic data in future studies to further elucidate this association.

Our study has certain limitations. Being a single-center observational study, there is a possibility of residual confounding, and caution should be exercised when applying these findings to other populations. Additionally, SUA levels were measured only once, and as SUA is a dynamic variable influenced by diet, this could introduce bias. The retrospective nature of our study limited our ability to control for all potential confounders, including lipid and cholesterol profiles, which are known to affect uric acid levels. Lastly, the relatively small sample size and the exclusion of most patients with secondary DCM to avoid the impact of comorbidities may have affected the generalizability of our results.

## Conclusion

In this single-center retrospective study, we observed that elevated SUA levels were independently associated with a significantly higher risk of all-cause mortality in children with DCM. This association was particularly pronounced in patients aged ≤10 years, indicating that age is a critical modifier of the relationship between SUA levels and prognosis in this population. Our findings suggest that SUA levels could serve as valuable prognostic biomarkers in pediatric DCM, especially for younger children. These results could aid in risk stratification and clinical decision-making for this challenging patient group.

## Data Availability

The original contributions presented in the study are included in the article/Supplementary Material, further inquiries can be directed to the corresponding author.
