# Supplementary material for: Uric acid elevation in pediatric patients with dilated cardiomyopathy and prediction of mortality
Source: Front Cardiovasc Med. 2024 Jul 23;11:1404755. doi: 10.3389/fcvm.2024.1404755 (PMC11301336; doi:10.3389/fcvm.2024.1404755)
Supplement: Supplementary file 1 [file Datasheet1.pdf]

SUPPLEMENTARY TABLES

Supplementary table 1 Univariate cox regression analysis of 1-year mortality in pediatric patients with DCM

Variable	HR (95% CI)	P value
**Age, per 1 year increase**	1.07 (1.02,1.13)	0.006
**Sex, male**	1.66 (0.87,3.17)	0.121
**BMI, kg/m^2**	1.12 (1.02,1.22)	0.015
**SCr, per 10μmol/L increase**	1.09 (1.03,1.16)	0.005
**BUN, mmol/L**	1.16 (1.07,1.25)	< 0.001
**eGFR, mL/min/1.73 m^2**	0.98 (0.96,1.01)	0.134
**LVEDD z-score, per 1**	1.0063 (0.8842,1.1454)	0.924
**LVEF, per 1% increase**	0.99 (0.96,1.02)	0.419
**NYHA/ROSS class, III/IV**	1.52 (0.84,2.75)	0.164
**Preexisting HF ≥ 6 months**	1.14 (0.53,2.45)	0.735

Abbreviations: HR hazard ratios, CI confidence intervals, BMI body mass index, NYHA New York Heart Association, SCr serum creatinine, BUN blood urea nitrogen, eGFR estimated glomerular filtration rate, LVEDD z-score left ventricular end diastolic diameter data were recorded and normalized into Z scores using age and body surface area, LVEF left ventricular ejection fractions

Supplementary table 2 Univariate cox regression analysis of overall mortality in pediatric patients with DCM

Variable	HR (95% CI)	P value
**Age, per 1 year increase**	1.11 (1.07,1.16)	< 0.001
**Sex, male**	1.32 (0.79,2.2)	0.289
**BMI, kg/m^2**	1.12 (1.03,1.22)	0.008
**SCr, per 10μmol/L increase**	1.11 (1.05,1.16)	< 0.001
**BUN, mmol/L**	1.15 (1.07,1.23)	< 0.001
**eGFR, mL/min/1.73 m^2**	0.98 (0.97,1)	0.095
**LVEDD z-score, per 1**	0.9946 (0.8925,1.1085)	0.923
**LVEF, per 1% increase**	0.97 (0.94,0.99)	0.016
**NYHA/ROSS class, III/IV**	1.45 (0.87,2.41)	0.154
**Preexisting HF ≥ 6 months**	1.26 (0.66,2.43)	0.48

Abbreviations: HR hazard ratios, CI confidence intervals, BMI body mass index, NYHA New York Heart Association, SCr serum creatinine, BUN blood urea nitrogen, eGFR estimated glomerular filtration rate, LVEDD z-score left ventricular end diastolic diameter data were recorded and normalized into Z scores using age and body surface area, LVEF left ventricular ejection fractions

Supplementary tables 3 The collinearity diagnostic analysis of overall mortality in pediatric patients with DCM

Variable	Age	Sex	SUA	SCr	BUN	BMI	LVEF
**Tolerance**	0.450	0.989	0.518	0.337	0.587	0.844	0.921
**VIF**	2.224	1.011	1.931	2.109	1.703	1.185	1.086

SUA, serum uric acid; SCr, serum creatinine; BUN, blood urea nitrogen; BMI body mass index; LVEF, left ventricular ejection fraction
